# Eosinophilic myocarditis: diagnostic pitfalls and therapeutic challenges. A Case Series

**DOI:** 10.3389/fcvm.2026.1818565

**Published:** 2026-05-13

**Authors:** A. S. Giordani, C. Menghi, A. Baritussio, F. Scognamiglio, C. Vicenzetto, M. Castegnaro, R. Marcolongo, G. Toscano, F. Chieco Bianchi, L. Iorio, R. Padoan, M. De Gaspari, S. Rizzo, C. Basso, A. L. P. Caforio

**Affiliations:** 1 Cardiology and Cardioimmunology Laboratory, Department of Cardiac Thoracic Vascular Sciences and Public Health, University of Padova, Padova, Italy; 2 Cardiac Surgery, Department of Cardiac Thoracic Vascular Sciences and Public Health, University of Padova, Padova, Italy; 3 Respiratory Medicine, Department of Cardiac Thoracic Vascular Sciences and Public Health, University of Padova, Padova, Italy; 4 Rheumatology, Department of Medicine, University of Padova, Padova, Italy; 5 Cardiovascular Pathology, Department of Cardiac Thoracic Vascular Sciences and Public Health, University of Padova, Padova, Italy

**Keywords:** anti–IL-5 monoclonal antibodies, endomyocardial biopsy, eosinophilic granulomatosis with polyangiitis, eosinophilic myocarditis, hypereosinophilic syndromes

## Abstract

**Background:**

Eosinophilic myocarditis (EM) is a rare and potentially life-threatening inflammatory heart disease. Diagnosis is frequently delayed because clinical presentations are heterogeneous, early cardiovascular magnetic resonance (CMR) lacks a pathognomonic pattern, and endomyocardial biopsy (EMB) is often performed late or after glucocorticoid exposure. A further pitfall is assuming that EM can be excluded in the absence of peripheral eosinophilia or overt extracardiac disease. Delayed recognition may result in irreversible myocardial injury, endomyocardial fibrosis and adverse clinical outcomes.

**Case summary:**

We report four cases of EM managed at our institution, each exemplifying a distinct diagnostic and therapeutic challenge. The first case was initially misclassified as apical hypertrophic cardiomyopathy on transthoracic echocardiography and CMR due to apical pseudo-hypertrophy, later evolving into endomyocardial fibrosis with severe mitral regurgitation requiring surgical annuloplasty, without clinical evidence of systemic disease. The second case was consistent with anti-neutrophil cytoplasmic antibodies (ANCA)-negative EGPA complicated by myocarditis; prolonged glucocorticoid exposure likely attenuated peripheral eosinophilia and masked eosinophils on EMB. The third case was initially classified as lymphocytic myocarditis based on an EMB performed under glucocorticoids, with eosinophilic infiltration and Loeffler endocarditis uncovered on repeat biopsy after steroid tapering. The fourth case presented with ST-segment elevation myocardial infarction secondary to coronary embolism in the setting of idiopathic hypereosinophilia. Across cases, we detail the diagnostic workup, emphasizing the complementary role of multimodality imaging and EMB, and outline therapeutic strategies, combining conventional immunosuppression with emerging targeted therapies against the interleukin-5 pathway.

**Conclusions:**

EM may present as an isolated, organ-specific cardiac disease in the absence of extracardiac involvement or peripheral eosinophilia, with a time-dependent prognosis. Diagnostic pitfalls frequently arise from reliance on non-invasive imaging alone and from smoldering disease course, particularly when chronic glucocorticoid therapy masks both clinical and histological features. Clinicians should maintain a high level of suspicion for EM diagnosis, even in absence of peripheral hypereosinophilia and if endomyocardial biopsy is obtained after acute or chronic steroid therapy, and consider possible thromboembolic complications, such as coronary artery embolization.

## 1 Introduction

Eosinophilic myocarditis (EM) is a potentially life-threatening inflammatory heart disease characterized by eosinophilic infiltration of the myocardium (1). Although traditionally regarded as a rare condition, EM is increasingly recognized across a broad etiological spectrum, including allergic or drug-induced reactions, infections, malignancies, and systemic eosinophilic disorders such as hypereosinophilic syndromes (HES) and eosinophilic granulomatosis with polyangiitis (EGPA), as well as an isolated, organ-specific immune-mediated entity (2, 3). Clinical presentations are highly heterogeneous, ranging from asymptomatic disease to fulminant myocarditis (4), yet the full clinical spectrum has not been systematically characterized to date. Despite advances in supportive care, short-term mortality remains substantial, with reported rates ranging from 19% to 55% in contemporary cohorts (2, 5, 6). Endomyocardial biopsy (EMB) represents the histological gold standard for diagnosis, while cardiac magnetic resonance (CMR) provides complementary non-invasive tissue characterization that supports diagnostic suspicion (7). However, CMR may be inconclusive at disease onset, as early-stage EM may lack pathognomonic features, thereby contributing to diagnostic delay (8). While early EMB has been associated with improved outcomes in fulminant myocarditis (9), the clinical impact of delayed histological diagnosis in non-fulminant myocarditis, particularly in rare forms such as EM, remains unexplored.

Once diagnosed, EM warrants prompt initiation of immunosuppressive therapy to prevent progression toward irreversible myocardial injury and adverse remodeling, ultimately leading to endomyocardial fibrosis (Loeffler's endocarditis) (10, 11). Conventional immunosuppressive regimens, primarily based on glucocorticoids with or without agents such as azathioprine, are non-targeted and frequently limited by toxicity or glucocorticoid dependence (6, 12, 13). In this context, targeted biologic therapies directed against eosinophil-mediated pathways, particularly anti–interleukin (IL)-5 or anti-IL5 receptor (IL5R) monoclonal antibodies, have demonstrated significant glucocorticoid-sparing efficacy in EGPA, HES, and other eosinophilic disorders (14–17). Although their use in EM remains off-label, emerging evidence suggests potential benefit in refractory or glucocorticoid-dependent myocardial eosinophilic inflammation (18, 19).

In this case series, we describe four patients with EM presenting with atypical phenotypes, including hypertrophic cardiomyopathy–like imaging features, occult eosinophilic myocardial infiltration masked by chronic glucocorticoid therapy, and acute ischemic presentations complicated by coronary embolism. In addition to describing complex scenarios related to this rare cardiac disease, our aim is to provide a clinically-relevant overview of diagnostic and therapeutic challenges in complex EM scenarios, with peculiar focus on possible pitfalls, as well as suggesting pragmatic strategies to optimize the management of EM in real-world practice (Table 1).

**Table 1:** Clinical, imaging, and histopathological features of four patients with eosinophilic myocarditis.

Variable	Case 1	Case 2	Case 3	Case 4
Age at diagnosis, Sex	36 y, F	60 y, F	81 y, M	28 y, F
Initial Presentation	Chest pain and worsening orthopnea; mild TnI elevation (400 ng/L), CRP 50 mg/L.	Acute dyspnea; markedly elevated TnI (4270 ng/L) and NT-proBNP (1744 pg/mL)	Progressive dyspnea; elevated TnI (360 ng/L) and NT-proBNP (11,703 pg/mL)	Chest pain; TnI 1900ng/L.
Systemic Eosinophilic Manifestations	Absence of systemic features.	ANCA negative EGPA with late-onset asthma, CRSwNP, glucocorticoid-responsive cutaneous involvement	Eosinophilic asthma	iHES with asthma and migratory pulmonary infiltrates.
Peripheral Eosinophilia	Intermittent mild eosinophilia (2.18 × 10⁹/L)	Previously episodic; normalized under therapy.	Peak 8.05 × 10⁹/L; normalized under therapy.	Intermittent, up to 1.8 × 10⁹/L; normalized under therapy.
Time from cardiac symptom onset to EM diagnosis	6 months	1 month	35 months (2.9 years)	65 months (5.4 years)
ECG at presentation	Sinus rhythm, anterior–inferior TWI	Sinus rhythm, within normal limits	Sinus rhythm, isolated PVC, and diffuse T-wave flattening	Sinus rhythm with TWI in V4-V6, followed by ST-segment elevation in inferior leads.
Imaging Findings at presentation (Echo and/or CMR)	Apical wall thickening; diffuse subendocardial LGE; myocardial edema; LV apical thrombus	Severe biventricular dysfunction (LVEF 30%); septal and apical myocardial edema; diffuse subendocardial LGE	Moderately reduced LVEF (40%) with apical hypokinesis and LV apical thrombus	Severely reduced LV systolic function (LVEF 31%) with two mobile apical thrombi; no myocardial edema or LGE at initial CMR.
EMB Findings	First biopsy: lymphomonocytic infiltrate with necrosis, rare eosinophils; viral PCR negative. Second biopsy: endomyocardial fibrosis.	Multifocal lymphomonocytic infiltrate with necrosis and granulomatous focus; no eosinophils (on glucocorticoids); viral PCR negative	**First biopsy**: active lymphomonocytic myocarditis without eosinophils (on glucocorticoids); viral PCR negative. **Second biopsy**: eosinophilic myocarditis, endocardial thrombosis (Loeffler endocarditis); viral PCR negative.	Active lymphomonocytic myocarditis with focal necrosis; eosinophils absent (on glucocorticoids).
Immunosuppressive/ Immunomodulant Therapy	High-dose glucocorticoids plus azathioprine until February 2022	Glucocorticoids followed by mepolizumab 100 mg/month, with subsequent glucocorticoid withdrawal.	Glucocorticoids plus Mepolizumab 300 mg/month, with subsequent glucocorticoid withdrawal.	Glucocorticoids plus Mepolizumab 300 mg/month, with subsequent glucocorticoid withdrawal.
Last Follow-up and Outcomes	Preserved LV function (LVEF 66%), minimal residual mitral regurgitation, NYHA class I, NT-proBNP 293 pg/mL	Improved LV function (LVEF 50%), NYHA class I, NT-proBNP 640 pg/mL.	Improved LV function (LVEF 54%), NYHA class I. Normal TnI and NT-proBNP.	Sustained remission with normal LV function (LVEF 64%), NYHA class I. Normal TnI and NT-proBNP.

CMR, cardiac magnetic resonance; CRP, C-reactive protein; ECG, electrocardiogram; EGPA, eosinophilic granulomatosis with polyangiitis; EMB, endomyocardial biopsy; iHES, idiopathic hypereosinophilic syndrome; LGE, late gadolinium enhancement; LV, left ventricle; LVEF, left ventricular ejection fraction; NT-proBNP, N-terminal pro–B-type natriuretic peptide; NYHA, New York heart association functional class; PCR, polymerase chain reaction; PVC, premature ventricular contraction; STE, ST-segment elevation; TnI, cardiac troponin I; TWI, T-wave inversion.

## 2 Cases presentation

### 2.1 Case 1

#### 2.1.1 Background

A 36-year-old woman with no prior cardiovascular or extracardiac medical history presented in November 2019 with persistent retrosternal chest pain. Initial ECG showed diffuse concave ST-segment elevation. Laboratory tests revealed mildly elevated high-sensitivity troponin I (hs-TnI; peak 30 ng/L; upper reference limit 14 ng/L) and elevated C-reactive protein (CRP 50 mg/L; upper reference limit 5 mg/L), while eosinophil counts remained within the normal range. She was provisionally diagnosed with acute idiopathic pericarditis and treated with high-dose oral NSAIDs and colchicine, showing initial symptomatic improvement. Two weeks later, transthoracic echocardiography demonstrated normal left ventricular (LV) size and function with mild pulmonary hypertension (sPAP 34 mmHg).

#### 2.1.2 Diagnostic assessment and therapeutic intervention

In December 2019, she was re-evaluated for worsening orthopnea. Troponin I was 400 ng/L, and ECG showed anterior and inferior T-wave inversions. During hospitalization, echocardiography revealed apical LV hypertrophy, while coronary CT angiography excluded coronary artery disease. CMR confirmed apical wall thickening (max 13 mm) with preserved biventricular function and no late gadolinium enhancement (LGE), supporting an initial diagnosis of apical hypertrophic cardiomyopathy (HCM). She was discharged on metoprolol. Despite initial management, symptoms persisted, leading to re-hospitalization in May 2020. Coronary angiography was normal. Repeat CMR demonstrated diffuse subendocardial edema and LGE, as well as a left ventricular apical thrombus. With the concomitant appearance of peripheral eosinophilia (peak: 2.18 × 10⁹/L), the overall clinical and diagnostic picture became suggestive of eosinophilic myocarditis. Although EGPA was considered, it was deemed unlikely because the patient lacked key features of EGPA, including adult-onset asthma, chronic rhinosinusitis with nasal polyps (CRSwNP), systemic or multi-organ involvement, and any clinical, laboratory, or imaging signs suggestive of vasculitis. Therefore, the presentation was interpreted as an eosinophilic cardiac disease without evidence of systemic vasculitis at the time of evaluation. Nonetheless, an organ-limited eosinophilic disorder, potentially representing an organ-limited form within the EGPA spectrum, cannot be definitively excluded. EMB confirmed acute myocarditis with lymphomonocytic infiltrates, myocyte necrosis, and eosinophils, consistent with EM (EMB-proven diagnosis); viral PCR was negative. Immunologic testing revealed markedly elevated total IgE levels (15,056 mKU/L), positivity for anti–myeloperoxidase (MPO) antibodies (11.8 kIU/L, with a perinuclear staining pattern on immunofluorescence), and the presence of anti-heart antibodies (AHA) with an organ-specific pattern. High-dose glucocorticoids (1 mg/kg/day) were initiated, followed by azathioprine (initiated at 50 mg/day and uptitrated to 100 mg/day). TnI, NT-proBNP and eosinophils normalized, and clinical status improved. A follow-up CMR in September 2020 showed partial resolution of myocardial edema but persistent subendocardial LGE, extending to the papillary muscles, with features consistent with evolving endomyocardial fibrosis. Anticoagulation was discontinued at that time.

#### 2.1.3 Follow-Up and outcome

Consecutive cardiac imaging surveillance revealed progressive worsening of mitral regurgitation. In May 2022, the patient was re-admitted with acute decompensated heart failure; transesophageal echocardiography confirmed severe functional mitral regurgitation, with leaflet tethering and annular dilatation. Right heart catheterization demonstrated elevated biventricular filling pressures with a preserved cardiac index. CMR showed extensive endocardial fibrosis without evidence of intracavitary thrombus, while repeat EMB demonstrated marked endocardial fibrous thickening, in the absence of active inflammation or myocyte necrosis, findings consistent with endomyocardial fibrosis as a sequela of prior eosinophilic myocarditis. She was then referred for surgical correction. In November 2022, the patient underwent successful mitral valve repair with semi-rigid ring and A3–P3 commissuroplasty, achieving optimal surgical results. Postoperative recovery was uneventful. Long-term follow-up through 2024 showed stable LV size and function (LVEF 66%), minimal residual mitral regurgitation, and reduced NT-proBNP levels (293 pg/mL). Mild eosinophilia recurred intermittently (0.99 × 10⁹/L) without clinical relapse. She remains asymptomatic (NYHA class I) at last follow-up.

### 2.2 Case 2

#### 2.2.1 Background

A 60-year-old woman with a history of late-onset asthma, CRSwNP, and recurrent glucocorticoid-responsive pruritic cutaneous rash presented in September 2021 with acute dyspnoea at rest. Her medical history was notable for episodic peripheral eosinophilia (since 2015, ranging between 1.0–1.5 × 10⁹/L) and repeated cycles of systemic glucocorticoids since 2014. Previous testing for antineutrophil cytoplasmic antibodies (ANCA) was negative. At admission, her medications included oral glucocorticoids, inhaled bronchodilators, and standard maintenance therapy for asthma. Chest radiography revealed bilateral pulmonary consolidations highly suggestive of eosinophilic pneumonia. Laboratory assessment showed markedly elevated high-sensitivity troponin (4270 ng/L) and NT-proBNP (1744 pg/mL), as well as mild peripheral eosinophilia (1.0 × 10⁹/L).

#### 2.2.2 Diagnostic assessment and therapeutic intervention

Transthoracic echocardiography demonstrated severely reduced LVEF (35%) with a mild circumferential pericardial effusion, not hemodynamically significant. Coronary angiography excluded obstructive coronary artery disease. CMR revealed severe biventricular dysfunction (LVEF 30%), with septal and apical myocardial oedema and diffuse subendocardial LGE, findings compatible with EM (CMR-proven EM). EMB demonstrated multifocal lymphomonocytic infiltrates with myocyte necrosis and a granulomatous focus containing rare giant cells; no eosinophils were identified, likely due to ongoing glucocorticoid therapy at the time of the procedure. Viral PCR testing was negative. The combination of upper and lower airway disease (late-onset asthma and CRSwNP), eosinophilia, systemic manifestations (skin rash), and eosinophilic/granulomatous myocardial involvement were considered highly suggestive of ANCA-negative EGPA, even in the absence of definite vasculitis in the sampled tissue. The patient received intravenous diuretics and non-invasive ventilation, resulting in rapid clinical improvement. Glucocorticoid therapy (1 mg/kg during the acute phase, subsequently tapered) was continued, and at discharge echocardiography showed improved LVEF of 46%.

#### 2.2.3 Follow-up and outcome

during follow-up, the patient reported persistent exertional dyspnoea but no limitation at rest. Cardiopulmonary exercise testing (C-PET) demonstrated moderately reduced functional capacity (peak VO₂ 12.9 mL/kg/min). Anti-heart antibodies (AHA) were negative. Mepolizumab (100 mg every 4 weeks) was initiated for severe eosinophilic asthma, facilitating glucocorticoids tapering; although higher dosing is commonly used in EGPA, treatment was individualized based on phenotype and response. Serial assessments from 2023 to 2025 showed clinical stability, significant weight loss, controlled asthma, and progressive reduction of NT-proBNP (from 1744 to 640 pg/mL). Repeat C-PET in April 2025 demonstrated improved peak VO₂ (14.6 mL/kg/min). At the last follow-up in November 2025, the patient was asymptomatic (NYHA I) on guideline-directed medical therapy (bisoprolol 7.5 mg daily, sacubitril/valsartan 49/51 mg twice daily, spironolactone 25 mg daily, dapagliflozin 10 mg daily) and mepolizumab (100 mg monthly), with no significant arrhythmias on Holter monitoring and an LVEF of 50% at echocardiogram.

### 2.3 Case 3

#### 2.3.1 Background

An 81-year-old man with a past medical history notable for late-onset eosinophilic asthma and CRSwNP presented in November 2019 with vomiting and diarrhea. Laboratory tests revealed marked peripheral eosinophilia (10.54 × 10⁹/L) and elevated troponin I (437.0 ng/L).

#### 2.3.2 Diagnostic assessment and therapeutic intervention

Initial multidisciplinary work-up excluded secondary and clonal causes of hypereosinophilia: ANCA testing, stool cultures, serum immunofixation, total IgE, and molecular screening for myeloid/clonal eosinophilic disorders (PDGFRB, FGFR1, FIP1L1–PDGFRA, JAK2 V617F, BCR–ABL) were all negative. Chest CT showed only minimal bibasilar opacities. High dose glucocorticoids (1 mg/kg) therapy was then initiated. At that time, transthoracic echocardiography demonstrated moderately reduced LVEF (40%) and coronary angiography ruled out coronary artery disease. EMB, performed after glucocorticoids exposure and resolution of peripheral eosinophilia, revealed active lymphomonocytic myocarditis without eosinophilic infiltration. Viral PCR panel was negative. Troponin and eosinophil counts normalized rapidly, and the patient was discharged on a tapering glucocorticoid regimen. Between 2020 and 2021, the patient remained asymptomatic (NYHA I). Mild intermittent eosinophilia persisted (ranging between 0.6–0.8 × 10⁹/L), but serial troponin and NT-proBNP remained within normal limits. Transthoracic echocardiography documented a stable recovery of LV systolic function over time. No evidence of vasculitis, connective tissue disease, or clonal hematologic pathology emerged.

**Figure 1:**
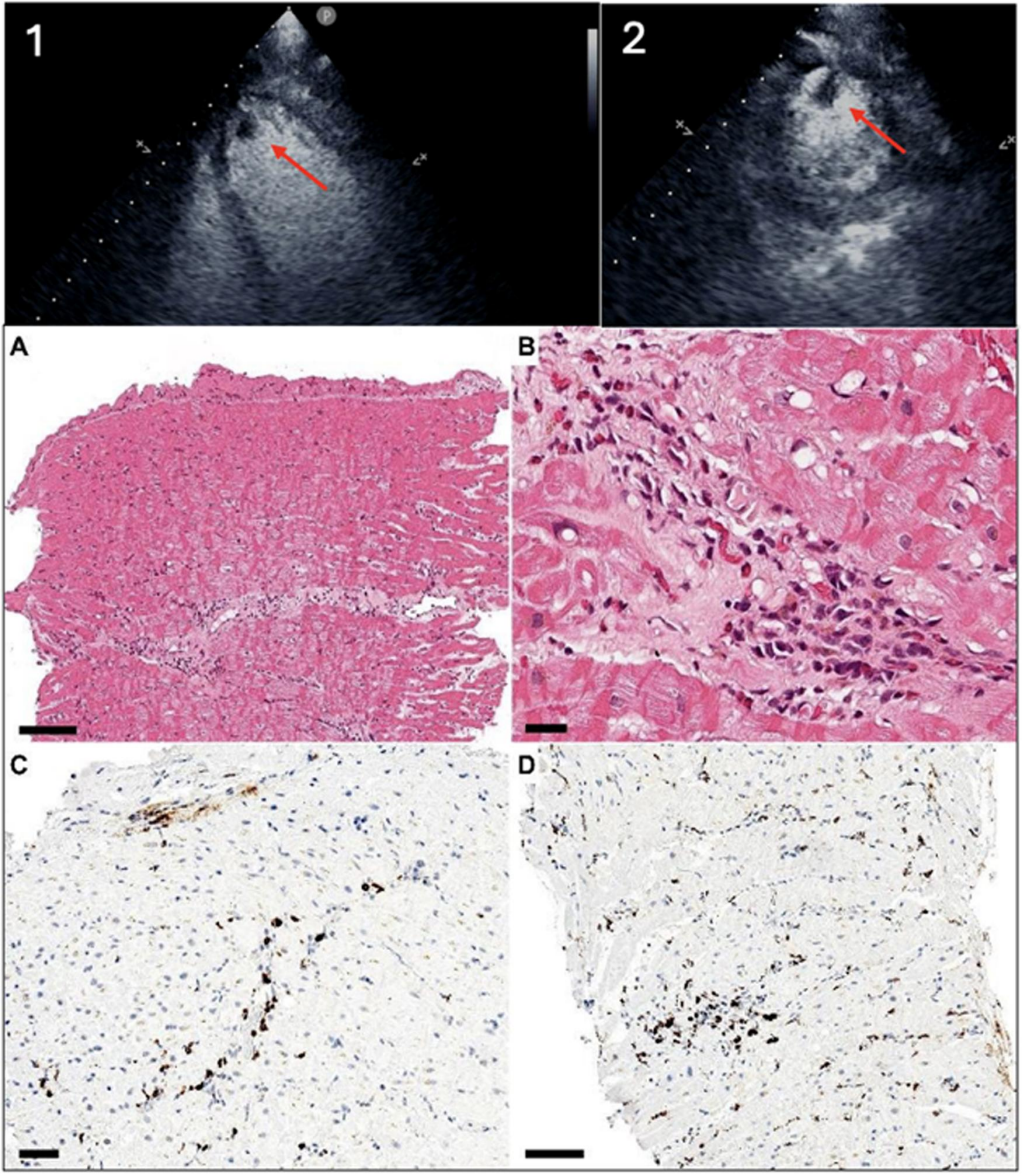
Echocardiographic and histological findings of case #3 diagnosed as eosinophilic myocarditis with endocardial thrombosis (loeffler endomyocarditis). Diagnostic findings of patient #3, who received a definitive diagnosis of eosinophilic myocarditis only during a second hospitalisation for recurrent myocarditis, occurring 3 years after the first episode. At presentation, transthoracic echocardiography showed left ventricular ejection fraction reduction (40%). Due to suspicion of intracavitary thrombus, Sonovue contrast was administered, confirming an apical left ventricular thrombus (panel 1: apical 4 chamber view, panel 2: focused parasternal short axis view, apical level; red arrows indicate the intraventricular thrombus, at the septal-apical level). Endomyocardial biopsy was repeated before initiating corticosteroid therapy and demonstrated eosinophilic myocarditis. In detail: Panels **A,B**) Multifocal eosinophilic infiltrates coupled with endocardial thrombotic deposition **(A)** and hemosiderin-rich macrophages **(B)** are evident at haematoxylin-eosin. Panels **C,D**) Immunohistochemistry showing scattered CD3-positive T lymphocytes **(C)** and aggregates of macrophages **(D)** Scale bars: A 100 µm, B 25 µm, C 50 µm, D 100 µm.

In October 2022, the patient was re-admitted with acute pulmonary oedema and new-onset atrial fibrillation with rapid ventricular response. Echocardiography revealed moderately reduced LVEF (40%) with apical hypokinesis and an LV apical thrombus; coronary angiography was normal. Repeat EMB demonstrated eosinophilic myocarditis (EMB-proven diagnosis) with features consistent with Loeffler endocarditis, including endocardial thrombotic deposition (Figure 1). Peak eosinophil count was 10.54 × 10⁹/L, while ANCA testing remained negative and IgE was modestly elevated (491 kUA/L). Overall, the combination of persistent hypereosinophilia and subsequent development of Loeffler endocarditis was most consistent with HES or idiopathic hypereosinophilia with cardiac involvement, in the absence of secondary or clonal causes. On this basis, mepolizumab 300 mg every 4 weeks was initiated in line with the approved HES regimen, with the aim of controlling eosinophilic activity and enabling glucocorticoid sparing. The patient was discharged in sinus rhythm on amiodarone therapy and anticoagulation (vitamin K antagonist, VKA).

#### 2.3.3 Follow-up and outcomes

From 2023 to 2025, the patient maintained clinical stability, with no angina or dyspnoea, and complete resolution of LV thrombus. NT-proBNP progressively decreased to 504 ng/L. Pulmonary function remained stable on Mepolizumab 300 mg monthly, with normal pulmonary function tests and suppressed eosinophilic airway inflammation (normal exhaled nitric oxide - FeNO). No further eosinophilic flares occurred after glucocorticoid discontinuation. At the last evaluation in December 2025, the patient remained asymptomatic (NYHA I), with preserved biventricular systolic function and no significant arrhythmias at Holter monitoring; Mepolizumab 300 mg monthly therapy was maintained and anticoagulation continued with direct oral anticoagulant (DOAC).

### 2.4 Case 4

#### 2.4.1 Background

A 28-year-old woman with no significant past medical history was admitted in March 2019 with persistent productive cough, migratory pulmonary infiltrates, and newly detected peripheral eosinophilia (3.0 × 10⁹/L). Glucocorticoids (prednisone 25 mg/day) were initiated for suspected eosinophilic lung disease, leading to rapid normalization of eosinophil counts. During hospitalization, she developed acute chest discomfort with marked TnI elevation (peak 1900ng/L), and negative T waves in V4-V6 on ECG. Transthoracic echocardiography revealed a moderately dilated LV with severely reduced systolic function (31%) and two mobile apical thrombi.

#### 2.4.2 Diagnostic assessment and therapeutic intervention

Cardiac MRI on day 8 showed LV dilation and apical thrombosis without clear signs of acute myocardial edema. Before scheduled EMB, the patient experienced recurrent chest pain, associated with inferior ST-segment elevation; emergent coronary angiography revealed complete thrombotic occlusion of a posterolateral branch of the right coronary artery in the absence of atherosclerosis. Successful percutaneous balloon angioplasty restored TIMI 3 flow, supporting myocardial infarction secondary to coronary embolism within the context of eosinophilic myocardial injury. After stabilization, EMB revealed chronic active lymphomonocytic myocarditis with focal necrosis but no detectable eosinophilic infiltrates, likely masked by ongoing glucocorticoid therapy. An extensive infectious, autoimmune and hematologic work-up, including JAK2, BCR-ABL1, PDGFRA/B, FGFR1, was negative and no alternative secondary trigger was identified. At discharge, prednisone was tapered to 6.25 mg/day, and eosinophil counts normalized (0.37 × 10⁹/L). Follow-up echocardiography (April 2019) showed complete resolution of LV thrombi and recovery of LVEF to 55%.

#### 2.4.3 Follow-Up and outcomes

Between 2019 and 2023, the patient experienced intermittent bronchospasm, recurrent eosinophilia (up to 0.9 × 10⁹/L), episodic chest discomfort, and fluctuating pulmonary infiltrates without cardiac dysfunction. In August 2024, she was re-hospitalized with recurrent chest pain, major troponin rise (hsTnI peak 6380 ng/L), and marked eosinophilia (1.8 × 10⁹/L). Cardiac MRI revealed preserved biventricular function with increased T1 mapping at the mid- and apical septum and increased apical T2 mapping, intramural LGE at the apical interventricular septum and focal subendocardial necrosis of the basal inferior wall; overall findings were consistent with active myocarditis, with mild septal apical edema and fibrosis, and sequelae of inferior embolic necrosis (CMR-proven diagnosis). Multidisciplinary evaluation led to a diagnosis of idiopathic hypereosinophilic syndrome (iHES) with cardiac, pulmonary, and vascular involvement. Mepolizumab 300 mg monthly was initiated, allowing glucocorticoid tapering and withdrawal. Over the following year, the patient achieved stable remission with normalization of eosinophil count, absence of further myocardial injury, and preserved biventricular function. Serial Holter monitoring demonstrated low-burden ventricular ectopy without sustained arrhythmias. At last follow-up (September 2025), she remained clinically stable (NYHA I) on mepolizumab (300 mg monthly), with normal troponin, NT-proBNP and eosinophils. The most recent echocardiography confirmed sustained recovery of LV function (LVEF 64%).

## 3 Discussion

EM represents a rare and potentially life-threatening form of myocarditis, characterized by heterogeneous clinical presentation, ranging from mild to fulminant presentations, thromboembolic events, and progression to endomyocardial fibrosis. Early recognition and prompt initiation of therapy are critical to prevent irreversible myocardial damage, including endomyocardial fibrosis. Our case series highlight recurring diagnostic pitfalls and practical management challenges, particularly when early imaging is non-specific, peripheral eosinophilia is absent or intermittent, or glucocorticoid exposure precedes histological sampling.

Each of the fours case, while maintaining its own individuality, shares overlapping clinical and diagnostic features with the others: case #1 and case #2 demonstrate possible phenocopies of EM (apical HCM in case #1, and non-ischemic dilated hypokinetic cardiomyopathy in case #2); cases #2, #3 and #4 show how EMB findings may be influenced by glucocorticoid therapy, demonstrating the importance of considering previous steroid therapy when interpretating histological results; finally, case #4 demonstrates the high thromboembolic risk in EM even during anticoagulant therapy for intracavitary thrombus, which was also diagnosed in cases #1 and #3.

### 3.1 Phenotypic mimicry and imaging challenges (case #1)

A key aspect emerging from the first case reported herein is the potential phenotypic overlap, particularly at EM onset, between eosinophilic myocarditis and other cardiomyopathies, i.e., apical hypertrophic cardiomyopathy, a phenomenon already reported in a limited number of cases, related to myocardial oedema during active myocarditis and/or apical endocavitary thrombus formation (8, 20, 21). In Case #1, both transthoracic echocardiogram and CMR first revealed apical wall thickening that closely mimicked apical hypertrophic cardiomyopathy, in the absence of other overt features suggestive of EM (i.e., absence of peripheral eosinophilia). As the disease evolved, subsequent imaging and histological assessment clarified the diagnosis, with progression to endomyocardial fibrosis and development of severe mitral regurgitation requiring surgical annuloplasty. Immunosuppressive therapy with glucocorticoids and azathioprine, administered according to the TIMIC protocol (13, 22), was well tolerated and contributed to clinical stabilization (23, 24).

The role of CMR in diagnosing myocarditis across heterogeneous and complex clinical presentations remains crucial. Recent evidence shows that CMR can uncover myocarditis even in patients initially managed as having myocardial infarction with non-obstructive coronary arteries (MINOCA) (25).

### 3.2 Histopathological pitfalls under glucocorticoid therapy (cases #2, #3 and #4)

Histopathology remains the diagnostic gold standard, although its sensitivity is limited by sampling error and can be further reduced when performed after glucocorticoid exposure. In our case series, initial histological confirmation of eosinophilic infiltration was not achieved in three of the four patients (cases #2, #3, and #4), with two showing lymphocytic and one polymorphic inflammatory infiltrate at the first EMB, likely due to ongoing high-dose glucocorticoid therapy at the time of biopsy (26, 27). Specifically, in case #2, chronic glucocorticoid use masked overt extracardiac eosinophilic manifestations at the initial cardiological evaluation (with only mild intermittent peripheral eosinophilia), whereas in case #3, EMB performed during glucocorticoid therapy initially led to a misdiagnosis, with eosinophilic infiltration becoming evident only after glucocorticoid tapering and repeat biopsy. This observation highlights how glucocorticoids may mask classical clinical and histopathological features, with direct clinical implication on both the optimal timing of EMB and the interpretation of its results. Additionally, these cases support a pragmatic approach: when clinical suspicion for EM remains high (e.g., compatible CMR pattern, intracavitary thrombus, systemic eosinophilic disease) but initial EMB is non-diagnostic, repeat EMB and integrated multidisciplinary reassessment should be considered.

### 3.3 Thromboembolic risk and anticoagulation (case #4)

Thromboembolic complications represent another major concern in EM, likely driven by endocardial injury, hypercoagulability, and ventricular dysfunction. In case #4, the patient experienced an ST-segment elevation myocardial infarction due to coronary embolism, despite ongoing high-dose glucocorticoid therapy, with intracavitary thrombi detected on imaging, possibly indicating that glucocorticoid therapy alone may be insufficient to prevent such events. Intraventricular thrombi were also observed in cases #1 and #3, and all were managed with VKA. Given the absence of EM-specific anticoagulation guidance, anticoagulant choice and duration were individualized (28–31). This experience reinforces the need to actively search for intracavitary thrombi and to consider prompt anticoagulation when thrombus is present, while acknowledging that optimal strategy and duration remain uncertain.

### 3.4 Therapeutic implications

An interesting aspect of our findings is that three of the four patients required long-term treatment with monoclonal antibodies (mepolizumab) due to a concomitant systemic eosinophilic disorder (EGPA, eosinophilic asthma or iHES). This allowed for glucocorticoid withdrawal and provided substantial clinical benefit, namely improved control of systemic eosinophilic symptoms and, importantly, favourable myocardial response: no relapses of EM were observed, and significant improvements were documented in NYHA functional class, LVEF at last follow-up, and cardiac biomarker. Importantly, no treatment discontinuation due to adverse effects was observed, indicating that these targeted strategies are not only effective but also well tolerated. These findings are consistent with the limited available case reports and small series documenting both the safety and myocardial response to anti–IL-5/5R biologics (24, 32–34). However, our case series should be interpreted as hypothesis-generating observations. In a small uncontrolled case series, improvements likely reflect the combined effects of background heart failure therapy, as well as prior or concurrent immunosuppressive therapy. Prospective studies coming from larger, homogeneous cohorts are needed to verify the efficacy and safety profile of biologic treatment in EM.

## 4 Conclusions

EM is a rare and underrecognized condition with heterogeneous clinical presentation. Non-invasive diagnostic modalities may fail to detect early or atypical forms, highlighting the critical role of timely EMB and the need to maintain high clinical suspicion, especially in patients with systemic eosinophilic involvement. Timely multidisciplinary evaluation and a multimodality diagnostic strategy, integrating CMR with appropriately timed EMB, repeated when necessary, may reduce diagnostic delays and help prevent irreversible remodeling and thromboembolic complications. Emerging therapies, such as anti–IL-5/5R monoclonal antibodies, show efficacy and a favorable safety profile, either alongside or instead of conventional immunosuppression, and may improve outcomes when integrated into a multidisciplinary management approach.

## Data Availability

The original contributions presented in the study are included in the article/Supplementary Material, further inquiries can be directed to the corresponding author.
